# AI: Can It Make a Difference to the Predictive Value of Ultrasound Breast Biopsy?

**DOI:** 10.3390/diagnostics13040811

**Published:** 2023-02-20

**Authors:** Jean L. Browne, Maria Ángela Pascual, Jorge Perez, Sulimar Salazar, Beatriz Valero, Ignacio Rodriguez, Darío Cassina, Juan Luis Alcázar, Stefano Guerriero, Betlem Graupera

**Affiliations:** 1 Department of Obstetrics, Gynecology, and Reproduction, Hospital Universitari Dexeus, 08028 Barcelona, Spain; 2 Department of Obstetrics and Gynecology, Clínica Universidad de Navarra, 31008 Pamplona, Spain; 3 Department of Obstetrics and Gynecology, University of Cagliari, 09042 Cagliari, Italy

**Keywords:** breast cancer, artificial intelligence, computer-aided diagnosis, breast biopsy, breast ultrasound

## Abstract

(1) Background: This study aims to compare the ground truth (pathology results) against the BI-RADS classification of images acquired while performing breast ultrasound diagnostic examinations that led to a biopsy and against the result of processing the same images through the AI algorithm KOIOS DS ^TM^ (KOIOS). (2) Methods: All results of biopsies performed with ultrasound guidance during 2019 were recovered from the pathology department. Readers selected the image which better represented the BI-RADS classification, confirmed correlation to the biopsied image, and submitted it to the KOIOS AI software. The results of the BI-RADS classification of the diagnostic study performed at our institution were set against the KOIOS classification and both were compared to the pathology reports. (3) Results: 403 cases were included in this study. Pathology rendered 197 malignant and 206 benign reports. Four biopsies on BI-RADS 0 and two images are included. Of fifty BI-RADS 3 cases biopsied, only seven rendered cancers. All but one had a positive or suspicious cytology; all were classified as suspicious by KOIOS. Using KOIOS, 17 B3 biopsies could have been avoided. Of 347 BI-RADS 4, 5, and 6 cases, 190 were malignant (54.7%). Because only KOIOS suspicious and probably malignant categories should be biopsied, 312 biopsies would have resulted in 187 malignant lesions (60%), but 10 cancers would have been missed. (4) Conclusions: KOIOS had a higher ratio of positive biopsies in this selected case study vis-à-vis the BI-RADS 4, 5 and 6 categories. A large number of biopsies in the BI-RADS 3 category could have been avoided.

## 1. Introduction

Breast ultrasonography (BUS) technology is now very mature. Many papers have demonstrated that BUS may discover smaller and more numerous invasive breast cancers than mammography in selected screening scenarios [1,2]. Operator dependence, a main pitfall of BUS, may be overcome by teaching staff to perform whole-breast examinations or by using automated breast ultrasound equipment. In the diagnostic scenario, BUS is a widely used technique to complement mammography. However, reporting when expertise is not available and the well-documented lack of specificity of BUS remain problematic. Artificial intelligence (AI) should help overcome these problems [3] as it is expected to “learn” to find lesions and/or suggest a diagnosis as would a second reader, or it may simply provide new information to reach a diagnosis [4].

The KOIOS DS ^TM^ (KOIOS) AI software only analyzes the images provided by a BUS examination and rates the risk of malignancy of each image submitted using a similar scale to the BI-RADS classification. KOIOS reaches its rating by processing the data using unknown pathways created by a black box [5], which is so-called because no one knows how it processes the information to deliver its results. The version we used, KOIOS, was developed after providing 450,000 images to train the black box through machine learning. (No more explicit information was provided by the manufacturer). It has been approved by the Food and Drug Administration and the European Medicines Agency.

In this retrospective study, we will compare the results of the predictive values of the traditional semiology used by radiologists at our institution to this radically different process using pathology reports as the ground truth. This represents a first step in determining whether AI could help in our diagnostic process.

We use a scale similar to the BI-RADS 2016 classification to reflect our grade of suspicion for BUS findings as follows: BI-RADS 1 (B1), no BUS findings; BI-RADS 2 (B2), benign BUS findings; BI-RADS 3 (B3), findings should render less than 2% true positives (TP); BI-RADS 4a (B4a), 3% to 10% TP; BI-RADS 4b (B4b), 10% to 50% TP; BI-RADS 4c (B4c), 50% to 90% TP; and BI-RADS 5 (B5), 90% to 100% TP.

The KOIOS software ranks probability in the following way: benign (Kbe), less than 0.5% probability of malignancy (PoM), similar to B1 and B2; probably benign (Kpb), less than 2% PoM, equivalent to B3; suspicious (KSS), equivalent to B4a and B4b; and probably malignant (KPM), equivalent to B4c and B5. The probability of malignancy is also graphically depicted in a continuous line. This graphic line seems to reflect a direct linear relationship to malignancy but is not to be taken as such.

## 2. Materials and Methods

Almost 30,000 BUS examinations were performed during 2019 by 9 experienced radiologists (range 3–35 years) using diverse modern BUS equipment. At our institution, the following criteria apply: Women under 40 have a BUS if the gynaecologist so demands. All women over 40 must have a mammography; BUS is performed if the breast pattern is considered heterogeneous or very dense or is following breast patterns c and d as identified by the ACR classification. BUS may also be performed on petition from the gynaecologist. Women of any age with a palpable lump reported by the gynaecologist, the patient, or noticed by the mammography technologist during the pre-mammography clinical examination must have a BUS. If the BUS reveals a B3 image, cytology is performed at the radiologist’s discretion. If cytology renders a fibroadenoma with hyperplasia, simple hyperplasia, or a suspicious or carcinoma report, a large-needle biopsy is recommended. Some few cases with an absence of cellular material are also biopsied after revision by the Multidisciplinary Committee of Breast Pathology (MCBP). Some B3 findings lead to a large-needle biopsy due to patient or gynaecologist preference (no data recorded on this subject). All B4 or higher BUS findings are mandated to have a large-needle biopsy. Mammography B4 findings must also have a BUS (with emphasis on the suspicious breast areas and axilla), and, if an ultrasound finding is correlated to the mammographic suspicious finding, a BUS-guided large-needle biopsy is performed. A large-needle biopsy implies at least a 14-gauge core biopsy or a vacuum-assisted biopsy, subject to the decision of the radiologist who performs the biopsy.

We retrieved the results of all consecutive large-needle biopsies of the breast performed at our institution during 2019 from the pathology department. Of 609 results, 404 BUS-guided biopsies had a BI-RADS classification attributed by our staff radiologists during the diagnostic examination and were performed at our institution and are those included in this study. Of the remaining pathology results, some biopsies performed in-house were not included in the study because there was no diagnostic workup at our institution; therefore, no BI-RADS category was recorded in our database. All 91 biopsies performed under mammographic guidance were not included because there was no correlated US finding described, or the mammographic finding was not correlated uncontestably with the US finding (mainly only microcalcifications in both circumstances). Some patients were included more than once because additional biopsies of other known findings (mainly non-suspicious images classified BI-RADS 2) or previously undetected findings (positive BUS second look after MRI) were indicated for staging purposes after revision by the MCBP.

Four of the nine radiologists that rated the original reports participated in this retrospective study. We reviewed the examinations which led to the 404 biopsies and retrieved the BI-RADS breast category from the diagnostic examination. The radiologists were also tasked with selecting the best image that depicted the finding that led to the biopsy and best represented the BI-RADS category from the diagnostic examination. They also verified that it was the same finding that was biopsied. They then submitted the single image for KOIOS analysis. Throughout this process, they were not able to see the pathology report. Submitting the image only involved clicking on the image and adding some extra data: location and size. Entering the extra data was not instrumental to the analysis. The KOIOS software defined the square region of interest (ROI) around the suspect image and, when prepped, rendered a rating almost instantly. The image processing of a single image using KOIOS is extremely simple and fast. The results are very clearly displayed and include the category as well as the PoM estimation. In some cases, the square ROI did not automatically appear on the image but manually depicting it was easily done. The results of the BI-RADS classification and the KOIOS classification were then compared to the pathology report and cross-referenced. The pathology report only used the classifications benign or malignant (including pTis).

### Statistical Analysis

Continuous variables were described using means and standard deviations. For categorical variables, frequencies and percentages were used.

The predictive positive value was used to compare the performance between BI-RADS and KOIOS.

The row probability result estimated by KOIOS was used in ROC curve analysis to evaluate the accuracy to discriminate between malign and benign lesions, but due to the selection bias (we selected only biopsied lesions), we judged it better to use positive predictive values (PPV) instead.

The area under the curve was 0.79 for malignant lesions, but due to the selection bias (we selected only biopsied lesions), we judged it better to use positive predictive values (PPV) instead.

All the analyses were exploratory. No formal sample size calculation was performed.

## 3. Results

Of the four hundred and four biopsies, seven were performed on B0, B1 and B2 categories, of which only the B1 case (meaning no BUS findings) resulted in a breast cancer diagnosis. A review of this case demonstrated that the lesion was not initially detected during the diagnostic BUS (while searching for a slow-developing density described in the mammography report; mammograms were at the disposal of the BUS radiologist). At the time of the biopsy (and before performing the scheduled stereotactic biopsy), a new BUS examination detected the suspect area and a vacuum-assisted US-guided biopsy was performed. Because there was no image of the finding in the diagnostic assessment and therefore, no BI-RADS classification reflecting on it, this case was excluded, leaving 403 cases for further evaluation.

The mean age of patients was 49.2 years with a standard deviation (SD) of 11.2 years. Malignant pathology results were found in 197 cases (48.8%) and benign in 206 (50.9%).

**Figure 1:**
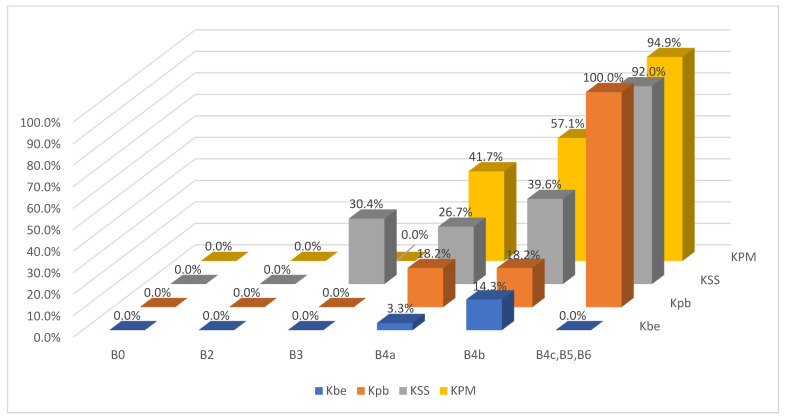
Probability of malignant biopsies for each combination of BI-RADS and KOIOS score.

The results of the BI-RADS and KOIOS classifications vis-à-vis the pathology report and their predictive values are presented in Table 1 and Table 2, respectively, and the cross-referencing of BI-RADS and KOIOS categories vis-à-vis the pathology results of these 403 cases can be seen in Table 3 (Figure 1).

Both B0 cases (estimated B3, low B4a images) were biopsied when previous examinations were not available after a holding period. Both were rated as KPM but had benign pathology results.

**Figure 2:**
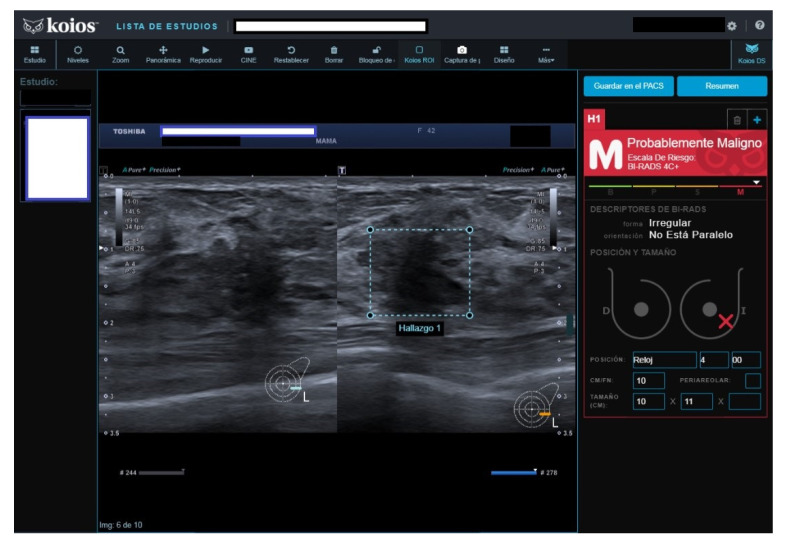
Suspicious image rated B2 by reader because of prior benign core biopsy (usual hyperplasia) and absence of change; rated KPM by KOIOS. Our multidisciplinary committee indicated a new and wider biopsy that was performed with US guidance and vacuum assistance. Pathology reported adenosis.

All four B2 cases had benign pathology results; a biopsy was performed for the following reasons after MCBP revision (B2 should not be biopsied): One had been core-biopsied 2 years previously, but due to the suspicious nature of the image, a wider vacuum-assisted biopsy was performed (Figure 2). The second had an unsuspicious scar from a previous malignant lumpectomy (micropapillary Ca), but because the patient presently had a cytologic papilloma diagnosis for nipple secretion, a new biopsy was performed. The third had a suspicious image from a previously benign biopsy (not performed at our institution) and previous studies with no obvious changes. Our MCBP recommended a new biopsy. The fourth had a suspicious mammography finding that was correlated with a benign-appearing BUS image. KOIOS reported cases one, two, and four as KPM and case three as Kpb.

**Figure 3:**
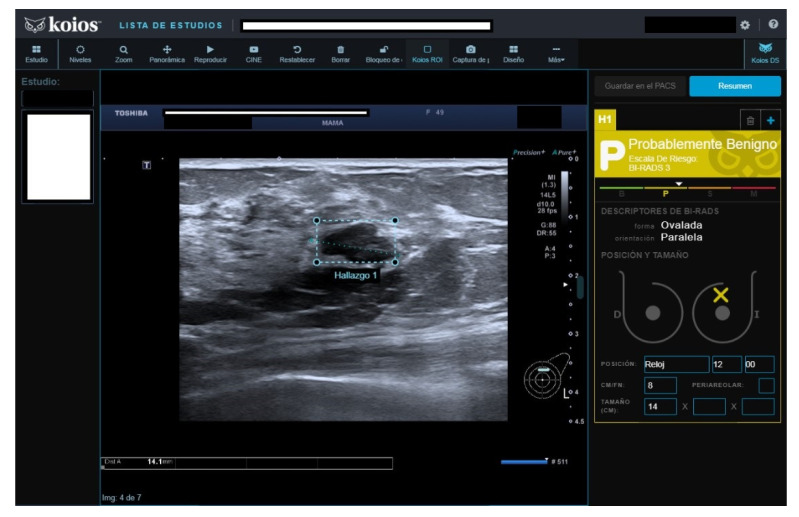
Nodule rated B3 by reader, Kpb by KOIOS. Cytology reported fibroadenoma with simple hyperplasia; biopsy rendered a fibroadenoma diagnosis.

**Figure 4:**
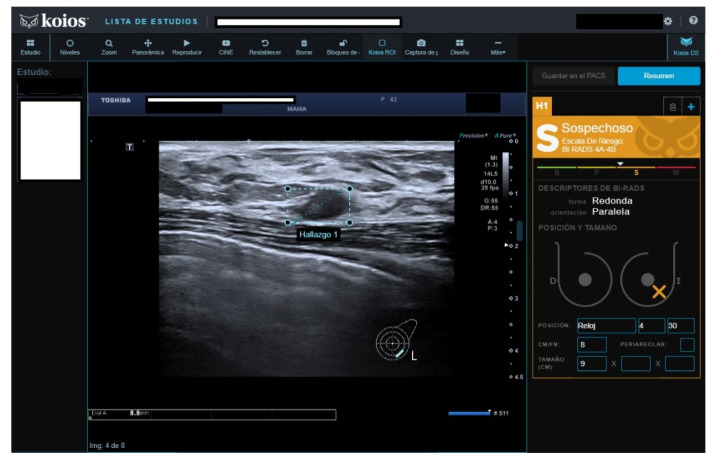
Nodule rated B3 by reader (no Doppler), KSS by KOIOS. Cytology reported grade 1 carcinoma. Biopsy confirmed invasive carcinoma.

Of fifty cases classified as B3, seven (15.4%) were malignant. The seven malignant cases were biopsied because cytology reported carcinoma in five, fibroadenoma with hyperplasia (FH) in one, and suspicious cytology in one. All seven were rated as KSS by KOIOS. The remaining 43 B3 cases with benign pathology results were mostly biopsied because of FH or simple hyperplasia cytology results (Figure 3). Two of the forty-three benign biopsies were for a B4b image in mammography correlated on BUS (one KSS, the other Kpb). Of the 43 B3 cases with benign biopsy results, KOIOS reported Kbe and Kpb in 26, and KSS (16) and KPM (1) in 17 (Figure 4). If KOIOS categories had been available and followed, only 24 biopsies (7 TP + 17 FP) would have been performed and no cancers would have been missed.

**Figure 5:**
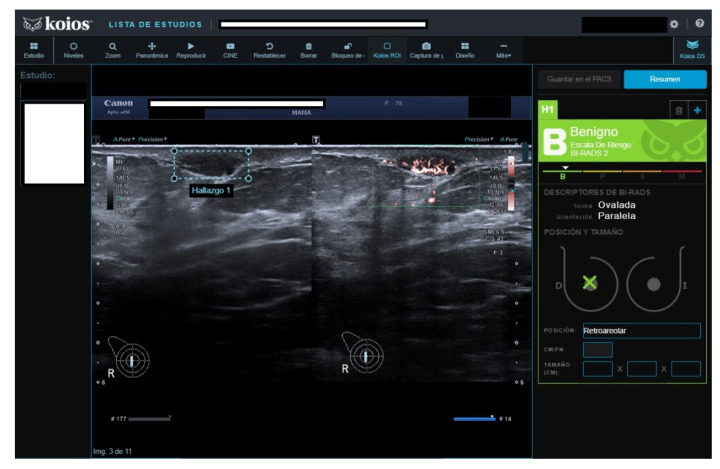
New nodule in right breast with previous carcinoma 25 years before. Rated B4a, Kbe by KOIOS. Surgery rendered a malignant fibrous histiocytoma.

There were 113 B4a cases, 24 with malignant biopsy results. Three of these twenty-four malignant results were in the Kbe or Kpb categories (Figure 5).

**Figure 6:**
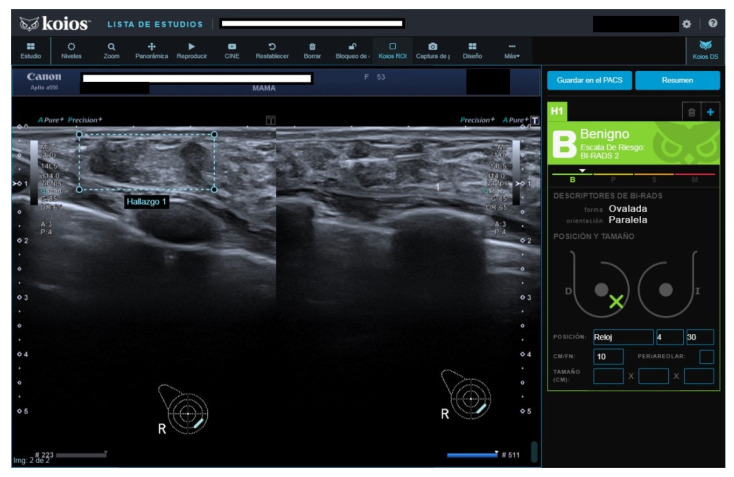
Palpable nodule rated B4b on BUS, Kbe by KOIOS. Confirmed invasive carcinoma.

There were 101 B4b category cases, 42 with malignant results. KOIOS reported seven benign and eleven probably benign cases; only one of the benign and two of the probably benign had a false negative result (Figure 6). There were 29 benign and 19 malignant KSS cases. The KPM cases numbered 35 and only 20 had a malignant pathology diagnosis.

The results of B3+B4a+B4b revealed 264 biopsies for 73 malignancies (27.7%), whereas Kpb+KSS rendered 220 biopsies for 96 malignant diagnoses (43.6%).

The BI-RADS 4c and BI-RADS 5 included one hundred and twenty-nine cases; nine of them were benign at pathology. Of these, one was in the Kbe category and the rest were in the KSS or KPM categories. Of the remaining one hundred and twenty B4c and B5 cases with malignant pathology results, KOIOS rated four as Kpb (3B4c and 1B5).

**Figure 7:**
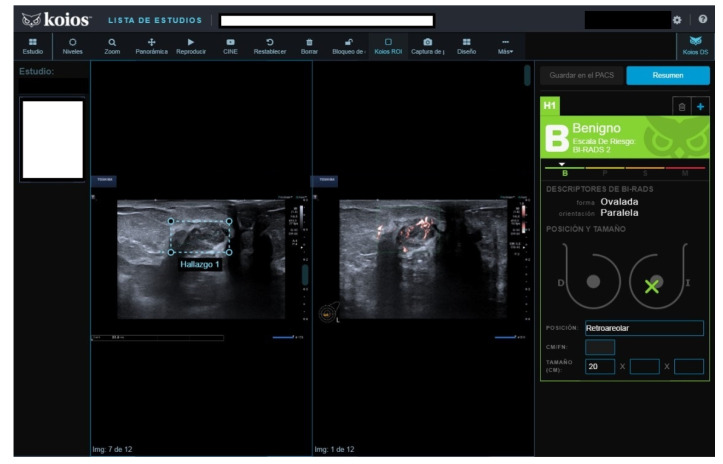
Rated B4c (B4b in mammography), KOIOS rendered a Kbe category. Pathology: chronic and acute inflammation. No signs of malignancy. Patient refused further surgery. BUS 2 years later found no significative residual lesion.

All B4, B5, and B6 347 biopsies were mandated; of these, 190 were malignant: 54.7% (190/347). KOIOS categories KSS and KPM should be biopsied: 312 biopsies would have rendered 187 malignant lesions (187/312: 60%), and 10 cancers would have been missed, two classified as benign and eight as probably benign by KOIOS (Figure 7).

These 10 cases corresponded to three pTis with associated mammographic suspicious microcalcifications (3 Kpb); one B4a with carcinoma cytology (Kpb); one malignant fibro histiocytoma (Kbe); three multicentric carcinomas, one with obvious mammographic correlation, the second with none at all and the third with palpable lesions (only one lesion image of each was analysed in this study); and two BUS findings rated B4c of 18mm (palpable, new) and 10mm (new), which KOIOS rated Kpb.

Four of the four cases with a BI-RADS 6 classification were malignant. KOIOS rated them KSS (3) and KPM (1). Three were rated malignant after a second look following an MRI and the fourth after a US biopsy of a second focus previously considered benign following a positive malignant diagnosis (confirmation of multicentric disease after a malignant diagnosis).

## 4. Discussion

We aimed to compare the BI-RADS classification of 403 biopsy pathology reports from our institution with the results of the analysis of biopsied images by KOIOS, a software application that categorizes US breast images through an artificial intelligence algorithm.

In breast diagnosis, the main question is whether to biopsy or not to biopsy a finding. Theoretically, B0 and B2 should not undergo core biopsy. Of our four hundred and three cases, six had these BI-RADS assignations; none were malignant. The two B0 were biopsied because previous examinations were not available after a holding period. KOIOS rated both as malignant. The four B2 cases were biopsied after review at our MCBP. KOIOS rated three as KPM and one as Kbe.

Cases rated as B4, B5, and B6 had a 54.7% positive predictive ratio. KOIOS KSS and KPM had a 60% predictive ratio but would have missed 10 cancers, most of which would have been biopsied anyway because of mammographic or clinical findings.

However, in our study conditions, KOIOS was clearly more successful at determining who had to have a biopsy for ratings B3+B4a+B4b. Our readers had 27.7% TP, but KOIOS Kpb+KSS, which are more or less equivalent to these BI-RADS ratings, rendered 43.6% TP. Of 50 B3 cases which had to have a biopsy due to our institutional criteria, KOIOS only suggested 24 biopsies, and no cancers would have been missed. It correctly rated all seven B3 lesions that were malignant as KSS. Cytology could have been avoided in 48 of 50 cases (two had suspicious mammography findings with benign-appearing BUS correlation and would have been biopsied anyway). In these 48 cases, KOIOS would have provided an immediate answer and any anguish felt by the patient while waiting for the cytology report and delayed biopsy would have been avoided. The thin line that separates B3 from B4a ratings is a critical breakpoint (short-term surveillance against biopsy). Our results suggest KOIOS could be of great help. Some reports indicate 20–30% TP results in breast biopsy series, largely because of very low PPV3 (positive predictive values for biopsy) for B4a images, which may lead to overdiagnosis and the wasting of resources and cause harm and unnecessary costs to women who undergo unnecessary biopsies [6]. On the other hand, for the time being, cases rated B4b and higher by a radiologist are always going to have a biopsy due to social, ethical, and legal priorities [7,8,9], notwithstanding the results of the KOIOS analysis.

Previous studies using KOIOS have reported on the influence of the AI software on the readers’ diagnosis process. Mango et al. assessed the impact of KOIOS on the diagnosis of 900 lesions. They concluded that it improved accuracy and decreased inter- and intra-observer variability [10]. At the SBIACR Breast Imaging Symposium 2020, Y. Gao analysed 200 cases, 155 of which were pathologically benign. He reported that associating KOIOS with the diagnostic process would have prevented 101 of 155 benign biopsies [11]. At the same symposium, J. Cavallo et al. presented a similar work to ours, which included 478 cases. As in our work, their data and conclusions showed that the software would have been most useful in the diagnosis of B4 (non-otherwise specified) and B4a-rated images with, respectively, 39/116 and 55/114 cases correctly labelled benign by KOIOS [12].

Important limitations to our study are the selection bias and retrospective design. We compared the readers’ BUS BI-RADS diagnosis to the KOIOS diagnosis using pathology results as ground truth, but only in cases that had been biopsied because of suspicious BI-RADS ratings or special circumstances. We do not know how KOIOS would have rated the 29,616 US examinations performed during 2019 and, especially, all B3 cases.

Lesser limitations are that KOIOS did not automatically deploy the ROI over the image in 22 of our cases. A study by Barinov et al. [13] found that the KOIOS category changes little between probably benign and suspicious when varying ROI boundaries (about 3% either way). We did not study this contingency in this study. Furthermore, as we only processed one image of each suspect lesion, we did not investigate whether different images of the same finding gave conflicting results.

How KOIOS would have affected our diagnosis was not studied. We share Barinov et al.’s [13] assertion that AI would be better used as a new but qualitatively different tool during the diagnostic process alongside the traditional semiology tools. As their study concludes, if used as a second reader, there is less predisposition to review the decision. This is not only because of a “case-closed” mindset, but also because if AI is the second reader, you cannot ask AI why it has reached a different conclusion; this is the “black box” dilemma [14].

For these same reasons, we do not think AI systems can be used in teaching diagnostics to unprepared practitioners because the same “black box” limitations apply.

## 5. Conclusions

We found that the KOIOS results are comparable to those of our breast-dedicated unit. KOIOS could be useful in places where the availability of an expert opinion is not at hand, not only in low- and middle-income countries but even in developed countries with underserved areas if trained non-medical operators perform a BUS on symptomatic women, to triage biopsy vs. non-biopsy [15,16,17]. However, we do not know with what material the black box was trained, or if it represents a universal teaching file for all human races and habitats. Furthermore, many of the lesions biopsied in our study were not clinically evident (no data on this issue); a fact that would not be usual in a triage setting. Therefore, our results are only significant for our single institution with a mostly European population.

We know of other AI software that have also had very positive results. Again, although some are produced by Asian firms and have been used in China, there are no global comparisons that we know of to measure their universal value. Most also place special emphasis on the B3-B4a conundrum, thus emphasizing this crucial point [18,19,20,21,22].

KOIOS has performed at least as well as the dedicated practitioners at our institution when deciding if a biopsy was warranted in these selected cases. For the time being, B4b and higher BI-RADS ratings must, for ethical, social, and medical responsibility reasons, still be biopsied if a human reader so decides. We look forward to seeing if, as suggested by our results, KOIOS can provide a better biopsy/no biopsy triage in B3 and B4a cases prospectively. The expectation that AI will keep demonstrating a fast-learning curve and raising the standard of care is promising.

## Figures and Tables

**Table 1:** BI-RADS.

	Total	Benign	Malignant	Predictive Value
B0	2	2	0	0%
B2	4	4	0	0%
B3	50	43	7	14%
B4a	113	89	24	21%
B4b	101	59	42	41%
B4c	53	6	47	88%
B5	76	3	73	96%
B6	4	0	4	100%
Total	403	206	197	

**Table 2:** KOIOS.

	Total	Benign	Malignant	Predictive Value
Kbe	52	50	2	3%
Kpb	39	31	8	9%
KSS	181	93	88	48%
KPM	131	32	99	75%
Total	403	206	197	

**Table 3:** Correlation of BI-RADS and KOIOS results against pathology diagnosis.

		Kbe				Kpb				KSS				KPM			
		TP	TN	FP	FN	TP	TN	FP	FN	TP	TN	FP	FN	TP	TN	FP	FN
	B *n* = 2															2	
**B0***n* = 2																	
	M																
	B *n* = 4						1									3	
**B2***n* = 4																	
	M																
	B *n* = 43		14				12					16				1	
**B3***n* = 50																	
	M *n* = 7									7							
	B *n* = 89		29				9					44				7	
**B4a***n* = 113																	
	M *n* = 24				1				2	16				5			
	B *n* = 59		6				9					29				15	
**B4b***n* = 101																	
	M *n* = 42				1				2	19				20			
	B *n* = 9		1									4				4	
**B4c, B5, B6***n* = 133																	
	M *n* = 124								4	46				74			

TP: true positive. TN: true negative. FP: false positive. FN: false negative. B0, B2, B3, B4a, B4b, B4c, B5 and B6 BI-RADS categories.

## Data Availability

Data are available upon reasonable request.
